# Study protocol: The DOse REsponse Multicentre International collaborative initiative (DO-RE-MI)

**DOI:** 10.1186/cc3718

**Published:** 2005-06-14

**Authors:** Detlef Kindgen-Milles, Didier Journois, Roberto Fumagalli, Sergio Vesconi, Javier Maynar, Anibal Marinho, Irene Bolgan, Alessandra Brendolan, Marco Formica, Sergio Livigni, Mariella Maio, Mariano Marchesi, Filippo Mariano, Gianpaola Monti, Elena Moretti, Daniela Silengo, Claudio Ronco

**Affiliations:** 1 Scientific Committee member; Leading Consultant, Anesthesiology Clinic, University of Düsseldorf, Germany; 2 Scientific Committee member; Director, Anesthesiology and Intensive Care Service, Hospital European Georges-Pompidou, Paris, France; 3 Scientific Committee member; Associate Professor, Department of Anesthesiology and Intensive Care, Medicine and Surgery Faculty, University of Milan, Italy; 4 Scientific Committee member; Director, Department of Anesthesiology and Intensive Care, Ospedale Niguarda, Milan, Italy; 5 Scientific Committee member; Vice-Head, Anesthesiology and Intensive Care Unit, Hospital Santiago Apostol, Vitoria, Spain; 6 Scientific Committee member; Vice-Head, Anesthesiology and Intensive Care Unit, Hospital Geral Sant Antonio, Porto, Portugal; 7 Steering Committee member; Epidemiology Consultant, Department of Nephrology, Hospital San Bortolo, Vicenza, Italy; 8 Steering Committee member; Vice-Head, Department of Nephrology, Hospital San Bortolo, Vicenza, Italy; 9 Steering Committee member; Director, Department of Nephrology, Hospital Santa Croce e Carle, Cuneo, Italy; 10 Steering Committee member; Director, Intensive Care Unit, Hospital G.Bosco, Torino, Italy; 11 Steering Committee member; Vice-Head, Intensive Care Unit, Hospital G.Bosco, Torino, Italy; 12 Steering Committee member; Vice-Head, Department of Anesthesiology and Intensive Care, Hospital Riuniti di Bergamo, Bergamo, Italy; 13 Steering Committee member; Vice-Head, Nephrology and Dialysis Unit, CTO Hospital, Turin, Italy; 14 Steering Committee member; Vice-Head, Department of Anesthesiology and Intensive Care,, Hospital Niguarda, Milan, Italy; 15 Steering Committee member; Vice-Head, Department of Anesthesiology and Intensive Care, Hospital Riuniti di Bergamo, Bergamo, Italy; 16 Steering Committee member; Vice-Head, Intensive Care Unit, Hospital G.Bosco, Torino, Italy; 17 Scientific Committee member; Director, Department of Nephrology, St. Bortolo Hospital, Vicenza, Italy

## Abstract

**Introduction:**

Current practices for renal replacement therapy in intensive care units (ICUs) remain poorly defined. The DOse REsponse Multicentre International collaborative initiative (DO-RE-MI) will address the issue of how the different modes of renal replacement therapy are currently chosen and performed. Here, we describe the study protocol, which was approved by the Scientific and Steering Committees.

**Methods:**

DO-RE-MI is an observational, multicentre study conducted in ICUs. The primary end-point will be the delivered dose of dialysis, which will be compared with ICU mortality, 28-day mortality, hospital mortality, ICU length of stay and number of days of mechanical ventilation. The secondary end-point will be the haemodynamic response to renal replacement therapy, expressed as percentage reduction in noradrenaline (norepinephrine) requirement. Based on the the sample analysis calculation, at least 162 patients must be recruited. Anonymized patient data will be entered online in electronic case report forms and uploaded to an internet website. Each participating centre will have 2 months to become acquainted with the electronic case report forms. After this period official recruitment will begin. Patient data belong to the respective centre, which may use the database for its own needs. However, all centres have agreed to participate in a joint effort to achieve the sample size needed for statistical analysis.

**Conclusion:**

The study will hopefully help to collect useful information on the current practice of renal replacement therapy in ICUs. It will also provide a centre-based collection of data that will be useful for monitoring all aspects of extracorporeal support, such as incidence, frequency, and duration.

## Introduction

The systemic inflammatory response syndrome is characterized by widespread endothelial damage caused by persistent inflammation from both infectious and noninfectious stimuli. The host employs hormonal and immunological mechanisms to counter the systemic inflammatory response syndrome. Hypoperfusion and shock result when homeostatic mechanisms are no longer able to keep the system in balance, leading to organ dysfunction [1].

Septic shock can be defined as sepsis with hypotension, despite adequate fluid resuscitation, along with evidence of perfusion abnormalities. It is the leading cause of acute renal failure (ARF) and mortality in intensive care patients. The pathogenesis usually involves a nidus of infection, which progresses to a bloodstream infection, followed by activation of mediators and eventual shock or multiorgan failure [2]. Both septic shock and severe bacterial infections are associated with increased levels of plasma cytokines such as tumour necrosis factor-α, IL-1, IL-6, IL-8 and IL-10, IL-1 receptor antagonist, and soluble tumour necrosis factor receptors types I and II. These mediators are produced in response to constituents of both Gram-negative and Gram-positive bacteria. Lipopolysaccharides of Gram-negative bacteria, and peptidoglycans, lipoteichoic acid and exotoxins of Gram-positive bacteria are largely responsible for the initial inflammatory cascade[3,4].

Various continuous and intermittent modalities of renal replacement therapy (RRT) are currently used. There has been slow acceptance of continuous RRT (CRRT) in intensive care units (ICUs) for the management of ARF, but this therapy is not new. In 1977 Kramer and coworkers [5] developed this technique following their accidental accessing of the femoral artery rather than the vein, creating an arterovenous circuit that yielded a very primitive but innovative approach. Problems with low blood flow and coagulation meant that this idea remained dormant for some time. It was not until the application of blood pumps and the substitution of arteriovenous with venovenous circuitry that the current practice of CRRT was born. In recent years remarkable advances in CRRT technology have been made, driven by nephrologists dedicated to improving efficiency and function. Today, however, intensivists are the most familiar with these techniques. Nevertheless, in some countries such as the USA, CRRT is still infrequently employed [6]. Other modalities include intermittent haemodialysis (IHD), slow extended daily dialysis [7], or daily haemodialysis [8]. Some of the reasons for the considerable variability worldwide in extracorporeal treatment of ARF include local practice (e.g. whether management is by nephrologists or intensivists), the centre's experience with the various techniques, organization and health resources. Various methods of extracorporeal treatment, whether intermittent or continuous, are currently being employed and no guidelines exist. This variability was highlighted in a recently completed observational study (the Beginning and Ending of Supportive Therapy for the Kidney [BEST Kidney] trial), which collected data on ARF management in 1743 patients in 54 ICU from 23 countries worldwide.

The practice of CRRT has apparently not changed, even following the prospective studies conducted by Ronco and coworkers [9]. Despite the positive findings of that prospective trial, the practice of a higher intensity CRRT has not been widely adopted into routine ICU practice. The most outstanding examples are Australia and New Zealand, where almost 100% of treatments are CRRT. A survey of several units active in the Australian and New Zealand Intensive Care Society Clinical Trials Group (Bellomo R, unpublished data, 2002) found that very few units had adopted the intensive CRRT regimen proposed by Ronco and coworkers [9]. Data from such Australian units shows instead that the vast majority (>90%) prescribe a 'fixed' standard CRRT dose of 2 l/hour, which is not adjusted for body weight. Thus, a 100 kg man would receive 20 ml/kg per hour – the dose shown to have the worst outcome in the study by Ronco and coworkers [9]. In another recent study that involved several Australian units (the BEST Kidney study), the median body weight for Australian patients was 80 kg, thus indicating that the vast majority receive a CRRT intensity of approximately 25 ml/kg per hour of effluent. Finally, although in the study conducted by Ronco and colleagues [9] the technique of CRRT was uniform in the form of continuous venovenous haemofiltration (CVVH) with postfilter fluid replacement, current practice includes a variety of techniques in addition to CVVH, such as continuous venovenous haemodialysis (CVVHD) and continuous venovenous haemodiafiltration (CVVHDF). Scarce information exists on the practice of CRRT in Europe, particularly regarding the actually delivered dose of therapy in critically ill patients with ARF (i.e. in those who could potentially derive more benefit from high volume convective therapy).

In a recent preliminary collaborative study [10] we reported that there was no significant difference between prescribed and delivered ultrafiltration rate (both in ml/min and in l/hour), which was related to the reduced down-time associated with the technique. However, of greater relevance is that the dose of dialysis was over 40 ml/kg per hour.

If we are to understand how dialysis doses are actually delivered in routine clinical practice in ICUs, an observational clinical study is needed to confirm how, to what extent and with what clinical indication the different modalities of RRT are administered. With this in mind we have initiated the DOse REsponse Multicentre International collaborative initiative (DO-RE-MI) trial. The primary end-point of DO-RE-MI is mortality (ICU mortality, 28-day mortality and hospital mortality), and the secondary end-point is the haemodynamic response to RRT, expressed as percentage reduction in noradrenaline (norepinephrine) requirement to maintain blood pressure.

## Materials and methods

**Figure 1:**
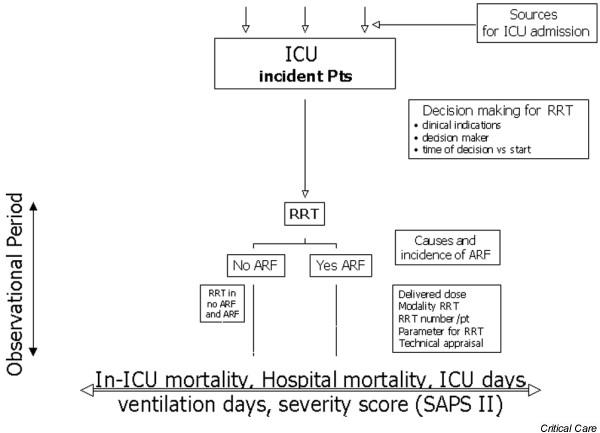
Flowchart of the DO-RE-MI observational study. All incident patients admitted to the intensive care unit (ICU) and requiring renal replacement therapy (RRT) will be followed up during RRT. At discharge, primary and secondary end-points will be recorded. All data will be entered in electronic case report form (CRF) and stored in a website [11]. The rectangles indicate the type of information that will be available from this study. ARF, acute renal failure; DO-RE-MI, DOse REsponse Multicentre International collaborative initiative; SAPS, Simplified Acute Physiology Score.

Figure 1 presents a study flowchart. Only incident patients with an indication for RRT will be recruited. The study is intended to describe current practices of RRT in all patients admitted to ICUs who are in need of RRT, with or without ARF. All data listed herein will be entered in electronic case report forms (CRFs) that are available via the internet [11]. The following rules will be applied without exception:

First, all patient data will be entered anonymously. To this aim, each centre will have a code, and patients will be consecutively assigned a unique number. Under no circumstances will there be any written or oral transmission of data that may make it possible to identify any patient. Failure to adhere to this will be followed by cancellation of the data from the website by the webmaster.

Second, data for each patient will be entered in a separate CRF. These data may be copied from paper CRFs in order to make the reporting of data from bed to computer station easier. All fields may be amended at any time until the patient's CRF is completed and closed. At this point, one may access the patients' CRF but it will be no longer be possible to amend the CRF. In the case of overt inconsistency, corrections must be detailed in writing (e-mail) by the person responsible for data quality for the centre or by the center itself. In all cases, no corrections will be permitted in the absence of an express written request. The person responsible for data quality will have access to the centre's CRF in printed form only. A registry will collect correspondence between the person responsible for data quality and the centre.

Third, completion of some fields in the CRF is mandatory. Failure to complete them will prevent progression to the following CRF and closure of the opened CRF. Failure to complete a CRF electronically will result in the patient being excluded from the study.

Finally, Each centre will be able to open CRFs for its own patients but never CRFs for patients from other centres.

### Case report form compilation

A guide to CRF compilation is presented in Table 1.

#### Case report form: Admission (step 1)

This CRF will automatically provide the patient's consecutive number. The user must enter the following data:

• sex,

• date of birth,

• weight,

• height,

• date/time of hospital admission,

• premorbid plasma creatinine levels,

• date/time of ICU admission,

• diagnosis at admission,

• Simplified Acute Physiology Score (SAPS) II (the index will be automatically calculated once each requested field is completed),

• Sequential Organ Failure Assessment (SOFA; the index will be automatically calculated once each requested field is completed).

#### Case report form: Criteria to initiate RRT (step 2)

This CRF will automatically provide the patient's consecutive number. The user must enter the date and time when the following clinical events (indexed numerically) occurred:

• 1. Oliguria (urine output <200 ml/12 hours),

• 2. Anuria (urine output <50 ml/12 hours),

• 3. High urea/creatinine,

• 4. Hyperkalaemia (>6.5 mmol/l or rapidly rising potassium),

• 5. Metabolic acidosis,

• 6. Fluid overload,

• 7. Hyperthermia (>41°C),

• 8. Immunomodulation,

• 9. What RIFLE (Risk Injury Failure Loss of function End stage renal disease) criteria [12] are applicable?

• 10. Others (to specify)

The user will also be asked to prioritize the criteria (from 1 to 3) when two or more specified. In addition, the modality chosen must be specified (defined as following and indexed numerically)

• 1. CVVH (as defined as ≤ 35 ml/kg per hour ultrafiltration rate in postdilution or <40 ml/kg per hour in predilution),

• 2. CVVHDF (defined as use of dialysate + replacement [define]),

• 3. High volume haemofiltration (defined as >35 ml/kg per hour in postdilution or >45 ml/kg per hour in predilution),

• 4. Pulse high volume haemofiltration (from 85 ml/kg per hour to 100 ml/kg per hour for 6–8 hours, followed by CVVH at 35 ml/kg per hour),

• 5. Coupled plasma filtration adsorption (CPFA) plus CVVH ,

• 6. IHD ('intermittent' includes conventional haemodialysis and slow extended daily dialysis, thereby encompassing all treatments in which sessions are separated from one another for 10 hours or more).

The CRF will then permit the user to specify any other relevent criteria, including the following:

• Staff problems,

• Technical problems,

• Product (e.g. fluids, lines, filters, machine) availability problems,

• Logistics,

• Others (to be specified).

#### Case report form: Modality-specific assessment time (step 3)

On the basis of the modality chosen in the 'Criteria to initiate RRT' CRF (see above), a specific CRF will be opened. The CRF for IHD will require data entry at baseline, at 4 hours and at treatment end. The CRF will automatically indicate the different visits (i.e. 0.0, 4.0, and treatment end). The CRF for **IHD **will request the following information:

• Decision taken (date/time),

• Start (date/time),

• Prescribed duration (only at assessment time 0),

• Delivered duration,

• Prescribed blood flow rate (only at assessment time 0),

• Delivered blood flow rate,

• Total weight loss (kg/session),

• Type of haemodialyzer (specify only commercial name),

• Surface of haemodialyzer (m^2^),

• Type of buffer (code number for lactate or bicarbonate),

• Anticoagulation (code number for heparin, citrate, prostacyclin, saline flushes, no anticoagulation),

• Arterial site of vascular access (code number for radial, femoral, pedidial, axillary access),

• Venous site of vascular access (code number for subclavian catheter, femoral catheter, jiugular, axillary catheter),

• Type of vascular access (code number for double lumen catheter, single lumen catheter),

• Vascular access gauge,

• Treatment interrupted (date/time),

• Resumption of treatment (specify date/time),

• End (day/time),

• Change in modality.

The CRF for **CVVH **will require data entry at 0 hours, at 1 hour, 3 hours, 6 hours, 12 hours, 24 hours, and every 24 hours thereafter and at treatment end. The CRF will automatically indicate the different assessment times (i.e. 0.0, 1.0, 3.0, 6.0, 12.0, 24.0, and so forth). Assessment times at 1.0, 3.0, 6.0 and 12.0 are optional, while assessment time at 24.0 and for multiples of 24 are mandatory. The following information will be requested:

• Decision taken (date/time),

• Start (date/time),

• Prescribed duration (only in assessment time 0),

• Delivered duration,

• Prescribed blood flow rate (only in assessment time 0),

• Delivered blood flow rate,

• Prescribed effluent (ml/hour; only at assessment time 0),

• Total effluent (ml/24 hours; only at assessment time 24 or last assessment time before treatment interruption/end),

• Prescribed reposition rate (ml/hours; only at assessment time 0),

• Total reposition (ml/24 hours; only at assessment time 24 or last assessment time before treatment interruption/end),

• Total volume removed from the patient (ml/24 hours),

• Type of haemodialyzer (as above),

• Surface (m^2^),

• Type of buffer,

• Anticoagulation (as above),

• Arterial site of vascular access (as above),

• Venous site of vascular access (as above),

• Type of vascular access (as above),

• Vascular access gauge,

• Treatment interrupted (date/time),

• Resumption of treatment (specify date/time),

• End (day/time),

• Change in modality.

The CRF for **CVVHD **(Note: I would suggest to indicate the modality in bold for clarity's sake) will require data entry at 0 hours, at 1 hour, 3 hours, 6 hours, 12 hours, 24 hours, and every 24 hours thereafter and at treatment end. The CRF will automatically indicate the different assessment times (i.e. 0.0, 1.0, 3.0, 6.0, 12.0, 24.0, and so forth). Assessment times at 1.0, 3.0, 6.0 and 12.0 are optional, while assessment times at 24.0 and for multiples of 24 are mandatory. The following information will be requested:

• Decision taken (date/time),

• Start (date/time),

• Prescribed duration (only in assessment time 0),

• Delivered duration (only at assessment time 24 or last assessment time before treatment interruption/end),

• Prescribed blood flow rate (only at assessment time 0),

• Dialysate (ml/24 hours),

• Effluent (ml/24 hours; only at assessment time 24 or last assessment time before treatment interruption/end),

• Total volume removed from patient (ml/24 hours; only at assessment time 24 or last assessment time before treatment interruption/end),

• Type of haemodialyzer (as above),

• Surface (m^2^),

• Type of buffer,

• Anticoagulation (as above),

• Arterial site of vascular access (as above),

• Venous site of vascular access (as above),

• Type of vascular access (as above),

• Vascular access gauge,

• Treatment interrupted (date/time),

• Resumption of treatment (specify date/time),

• End (day/time),

• Change in modality.

The CRF for **CVVHDF **will require data entry at 0 hours, at 1 hour, 3 hours, 6 hours, 12 hours, 24 hours, and every 24 hours thereafter and at treatment end. Assessment times at 1.0, 3.0, 6.0 and 12.0 are optional, while assessment times at 24.0 and for multiples of 24 are mandatory. The CRF will automatically indicate the different assessment times (i.e. 0.0, 1.0, 3.0, 6.0, 12.0, 24.0, and so forth). The following information will be requested:

• Decision taken (date/time),

• Start (date/time),

• Prescribed duration (hours; only at assessment time 0),

• Delivered duration (hours; only at assessment time 24 or last assessment time before treatment interruption/end),

• Prescribed blood flow rate (ml/min; only at assessment time 0),

• Prescribed effluent (ml/hour; only at assessment time 0),

• Delivered effluent (ml/ 24 hour; only at assessment time 24 or last assessment time before treatment interruption/end),

• Prescribed reposition rate (ml/hour),

• Delivered reposition rate (ml/24 hours; (only at assessment time 24 or last assessment time before treatment interruption/end),

• Dialysate (ml/24 hours; only at assessment time 24 or last assessment time before treatment interruption/end),

• Total volume removed from the patient (ml/24 hours; only at assessment time 24 or last assessment time before treatment interruption/end),

• Type of haemodialyzer (as above),

• Surface (m^2^),

• Type of buffer,

• Anticoagulation (as above),

• Arterial site of vascular access (as above),

• Venous site of vascular access (as above),

• Type of vascular access (as above),

• Vascular access gauge,

• Treatment interrupted (date/time),

• Resumption of treatment (specify date/time),

• End (day/time),

• Change of modality.

Independently of modality chosen, all 'Modality-specific assessment time' CRFs include the following additional fields:

• SOFA (full set of data; only at assemement time 24 and for multiples of 24),

• Creatinine,

• Urea,

• Na,

• K,

• White blood cells (10^3^/μl),

• Platelets (10^3^/μl),

• Hb,

• pH,

• PaO_2_,

• PCO_2_,

• Bicarbonate,

• FiO_2_,

• Body temperature,

• Urine volume,

• Fluid balance (only at assessment time 24),

• Bicarbonate,

• Fractional inspired oxygen,

• Urine volume (ml/24 hours),

• Fluid balance (ml/24 hours),

• Systolic blood pressure (mmHg),

• Diastolic pressure (mmHg),

• Mixed venous oxygen saturation,

• Heart rate,

• Cardiac output,

• Cardiac index

• Pulmonary artery pressure,

• Systemic vascular resistance index,

• Intravascular blood volume index,

• Extravascular lung water index,

• Stroke volume variation,

• Vasopressor administration (milligrams of vasopressors/previous 24 hours): adrenaline (μg/kg per min), noradrenaline (μg/kg per min), dobutamine (μg/kg per min), dopamine (μg/kg per min), vasopressin (units/previous 24 hours), terlipressin (mg/previous 24 hours),

• Vasodilator administration,

• Other treatments: steroids (mg/24 hours; specify what type), recombinant human activated protein C, antithrombin III, protein C,

• Coagulation: activated partial thromboplastin time (diff versus control), activated clotting time (diff versus control), INR (%),

• Factors complicating RRT: logistics, organization, vascular access, anticoagulation, circuit patency, haemodialyzer performance.

#### Guidelines given in the CRF

**Figure 2:**
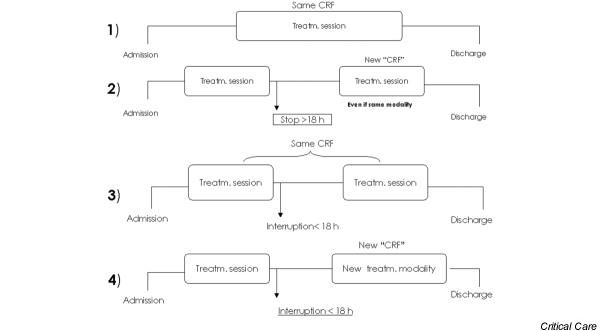
Examples of how the different case report forms will be applied. Four different cases are summarized, encompassing treatment interruption or end in relation to the compilation of case report forms (CRFs). Case 1 is the easiest case. The patient is admitted to the intensive care unit (ICU), is treated with renal replacement therapy (RRT), ends treatment and is discharged. The patient has a single CRF. In case 2 the patient is admitted and is treated with RRT, but this treatment is stopped for longer than 18 hours (this is defined as treatment end). However, the patient is later started on RRT again. A new CRF (even if the modality is the same) will need to be completed. In this case, the patient has two or more CRFs (as in the case of more than one treatment stoppages for longer than 18 hours). In case 3 the patient is admitted and is started on RRT, which is stopped for less than 18 hours (defined as interruption). The patient is then restarted and the compilation is continued on the same CRF. Case 4 is similar to case 3, with the important difference being related to the change in modality following treatment interruption. In this case, each change of modality will require a new CRF.

'Treatment interruption' is defined as when a treatment is stopped and resumed within 18 hours. In the case of treatment interruption the CRF will be continued and the treatment that follows will be considered in the context of the preceding one. The only exception is when, after RRT interruption, the modality is changed (see below under 'Case report form: Change modality (step 3)'; Fig. 2).

'Treatment end' is defined as when a given RRT is stopped because of clinical or other factors for more than 12 hours or when clinical or other factors have changed since the start of RRT. Should the patient be started on another RRT, then the latter shall be considered a new one.

In the case that the modality is changed, a new CRF will need to be filled in (see 'Criteria to change RRT'). This will be followed by a new CRF 'Modality-specific assessment time' (also see Table 1).

#### Case report form: 'Change to modality'

Each centre will be asked to define the clinical/practical reasons for changing a modality. The change to modality may be necessary after treatment is interrupted. In this case, the following treatment will be considered a new treatment. This CRF aims to provide information on why the modality was chosen. It is similar to the CRF: Criteria to initiate RRT.

#### Case report form: Outcome (step 4)

At discharge of the patient, the following information should be provided:

• SAPS II (all sets of data; previous 24 hours before discharge from ICU),

• SOFA (all set of data; previous 24 hours before discharge from ICU),

• IHD needed in ward (yes/no),

• Creatinine at discharge (μmol/l; mg/%),

• Urea at discharge (μmol/l; mg/%),

• In-ICU mortality (yes/no),

• Ventilation days (number of days),

• 28-day mortality (yes/no),

• Discharged from ICU (date),

• Discharged from hospital (date),

• Hospital survival (yes/no),

• Date of last RRT session.

### Calculation of dialysis dose

The dialysis dose will be calculated differently according to the type of modality. In the case of CVVH, solute transport is achieved by pure convection. The solute flux across the membrane is proportional to the ultrafiltration rate (Qf) and the ratio between the concentration of the solute in the ultrafiltrate and in plasma water (sieving coefficient S). For solutes freely crossing the membrane, S values are equal or close to 1. Because clearance is calculated from the product Qf × S, when S is proximal to 1, as for urea, clearance is assumed to be equal to Qf, provided that replacement solution is given in postdilution mode. For diffusive techniques (IHD or CVVHD), the clearances will be calculated on the basis of the delivered operational parameters on an experimentally constructed relationship (blood flow versus clearance) at three different dialysate flow rates (in CVVHD at 1 and 2 l/hour) for each given haemodialyzer. In mixed convective/diffusive techniques (e.g. CVVHDF), this relationship will constructed at two dialysate flows (1 and 2 L/hour) and at three ultrafiltration rates.

### Statistical analysis

#### Primary end-point

The power for a test of the null hypothesis (logistic regression, one continuous predictor) was calculated as follows. The one goal of the proposed study was to test the null hypothesis (i.e. that there is no relationship between clearance and event rate). Under the null condition, the event rate (0.51) is the same at all values of clearance or, equivalently, the odds ratio is 1.0, the log odds ratio (beta) is 0.0 and the relative risk is 1.0.

**Figure 3:**
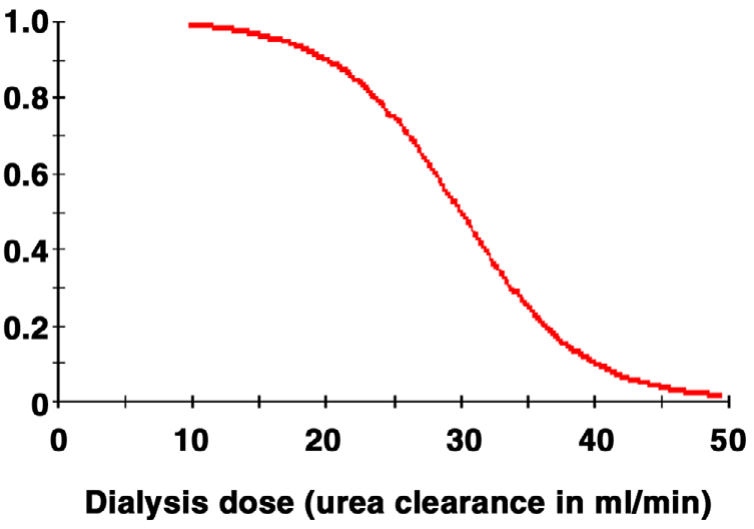
Mortality rate as a function of dialysis dose (expressed as urea clearance ml/min).

Power is computed to reject the null hypothesis under the following alternate hypothesis. For clearance values of 29.8 and 35.0, the expected event rates are 0.51 and 0.25. This corresponds to an odds ratio of 0.32, beta (log odds ratio) of -0.22, and a relative risk of 0.49. This effect was selected as the smallest effect that would be important to detect, in the sense that any smaller effect would not be of clinical or substantive significance. It is also assumed that this effect size is reasonable, in the sense that an effect of this magnitude could be anticipated in this field of research. In these computations, we assume that the mean clearance value will be 29.8 with a standard deviation of 10.0, and that the event rate at this mean will be 0.51 (Figure 3).

The sample size will be of a total of 110 patients.

**Figure 4:**
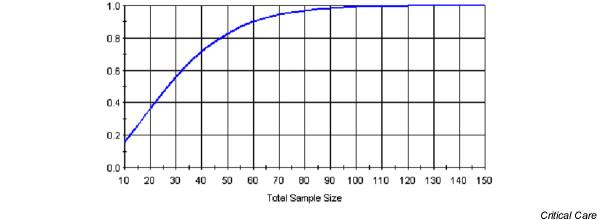
Power as a function of sample size.

Alpha and tails. The criterion for significance (alpha) has been set at 0.01. The test is two-tailed, which means that an effect in either direction will be interpreted (Figure 4).

Power. For this distribution (clearance mean of 29.8, standard deviation of 10.0), baseline (mean event rate of 0.51), effect size (log odds ratio of -0.22), sample size (*n *= 110) and alpha (0.01, two-tailed), the power is 1.00. This means that close to 100% of studies would be expected to yield a significant effect, rejecting the null hypothesis that the odds ratio is 1.0 [13-16]. The software used will be Epi Info (Utilities StatCalc Epi Info™ version 3.3, release date: 5 October 2004; Division of Public Health Surveillance and Informatics, Centers for Disease Control and Prevention, Atlanta, GA, USA) [17] and Power And Precision™ (version 2.0; release date: 20 December 2000) [18].

#### Secondary end-point

Based on data from one participating center (Milan Niguarda), approximately 20% of all RRT-treated patients have high noradrenaline requirements. A sample size of 27 patients will have 80% power to detect a difference in means of 0.295 (e.g. a mean of 2.5 μg/kg per min, assuming a standard deviation for differences of 0.600, using a paired t-test with a 0.05 one-sided significance level). Therefore, a minimun of 135 patients should be enrolled. Assuming a 20% dropout rate, the minimum number of patients to be recruited is 162.

### Study limitations

This will be an observational study. Based on the conventional meaning [19], an observational study cannot modify actual practice or therapy. In this study, the decision as to whether RRT should be commenced is at the discretion of the attending physician.

## Discussion

The practice of CRRT has been subject to much debate. Only a few prospective randomized studies have been performed and published on the relationship between CRRT and outcome, and so conclusions are difficult to draw [20,21]. As emphasized in a recent editorial [22], in the field of artificial organs, prospective observational studies, despite their inherent limitations, have been performed because they are more affordable but are also capable of providing useful information from practical and medical standpoints.

Guerin and coworkers [23] studied 587 patients requiring haemodialysis and followed them until hospital discharge. Among the 587 patients, 354 received CRRT and 233 intermittent RRT as first choice. CRRT patients had a greater number of organ dysfunctions on admission and at the time of ARF, as well as higher SAPS II. Mortality was 79% in the CRRT group and 59% in the intermittent RRT group. Logistic regression analysis showed decreased patient survival to be associated with SAPS II on admission, oliguria, admission from hospital or emergency room, number of days between admission and ARF, cardiac dysfunction at time of ARF, and ischaemic ARF. No underlying disease or nonfatal disease, and absence of hepatic dysfunction were associated with an increase in patient survival. The type of RRT was not significantly associated with outcome. Those authors concluded that RRT mode was not of prognostic value.

The largest observational study ever performed (the BEST Kidney) was recently completed and reported in part [24]. A total of 1743 consecutive patients, who either were treated with RRT (CRRT or IHD) or fulfilled predefined criteria for ARF, were studied. Importantly, the findings indicated a marked difference in mortality rates across the different ICUs, suggesting that the practice of RRT may yet exert an influence on mortality [Bellomo R, unpublished observation]. Increasing the dose to 35 ml/kg per hour would be associated with a significantly greater survival in all ARF patients. However, higher dialysis doses (45 ml/kg per hour) had no statistically significant impact in the ARF patients studied. However, in a subgroup analysis including only those patients with sepsis, there was a trend suggesting that this might be the case.

Despite the numerous publications that suggest a benefit from delivering higher dialysis doses (for review [25]), the real impact in critically ill patients is unclear. An observational clinical survey to evaluate what modality, for what reasons and what outcomes are important is needed if we are to understand how dialysis is delivered and at what dose in routine ICU practice; what the benefits, if any, are in terms of haemodynamics; and, finally, what are the benefits in terms of patient outcome as the primary end-point.

Current treatments for multiorgan dysfunction with ARF include many forms of CRRT that differ with respect to following factors: dose of dialysis, the extent of convection and diffusion, flow rates (blood, dialysate and replacement fluids) and anticoagulation protocols (heparin, citrate, flushes of saline). Ancillary to these factors are the choices of predilution or postdilution, of haemodialyser (surface, membrane) and of vascular access. It is still unknown whether and to what extent the prescribed dose comforms with evidence-based literature and, more importantly, how the delivered dose diverges from the prescribed one.

The present study, as indicated in the present protocol, should help to resolve at least some aspects of this still largely undefined area of critical care.

## Conclusion

The present study should provide insight into how RRT is currently practiced in ICUs and should hopefully provide answers to as yet undefined questions, such as the following: what are the criteria for beginning and ending treatment?; what is the currently delivered dose of dialysis?; how is fluid control taken care of?; what schedules are mostly used?; how is technology used (or not used)?; and, finally, what are the reasons for down-time in RRT? The ultimate goal will be to define how the dialysis dose actually delivered may impact on the outcome primary end-points of ICU patients.

## Key messages

• 	Choice of RRT in renal and nonrenal indications

• 	Delivered dose of dialysis and its impact on outcome measures (primary end-point)

• 	Hemodynamic response to RRT (secondary end-point)

• 	Causes for down-time in CRRT

## Abbreviations

ARF = acute renal failure; CRF = case report form; CRRT = continuous renal replacement therapy; CVVH = continuous venovenous haemofiltration; CVVHD = continuous venovenous haemodialysis; CVVHDF = continuous venovenous haemodiafiltration; ICU = intensive care unit; IHD = intermittent haemodialysis; IL = interleukin; RRT = renal replacement therapy; SAPS = Simplified Acute Physiology Score; SOFA = Sequential Organ Failure Assessment.

## Competing interests

The author(s) declare that they have no competing interests.

## Authors' contributions

The Scientific Committee comprised Kindgen-Milles D (Duesseldorf, Germany), Journois D (Paris, France); Fumagalli R (Bergamo, Italy), Ronco C (Vicenza, Italy), Vesconi S (Milan, Italy), Maynar J (Vitoria, Spain) and Marinho A (Porto, Portugal), who reviewed the different versions of the study protocol prepared by the Steering Committee and gave the final approval of the version to be published. The Steering Committee comprised the following individuals: Livigni S, Maio M (Torino, Italy), Marchesi M (Bergamo, Italy), Monti GP (Milano, Italy) and Silengo D (Torino, Italy), who made substantial contributions to conception and design and to establishing the CRF; Bolgan I (Vicenza, Italy) defined the way in which data will be analyzed and interpreted; Brendolan A (Vicenza, Italy), Formica M.(Cuneo, Italy), and Mariano F (Torino, Italy) helped in the definition of RRT modalities and reviewed the final case report forms.

## Figures and Tables

**Table 1:** Guide to case report form compilation

Step	Details
Step 1	Complete CRF: Admission

Press 'save' and open CRF: Criteria to initiate RRT	
Step 2	Complete CRF: Criteria to initiate RRT:
	• Indicate one or more criteria to initiate RRT and their priority score (from 1 [low] to 3 [high])
	• In patients with ARF, choose which RIFLE criteria are applicable
	• Indicate what you expect to happen using the technique you have chosen

Press 'save'; the next CRF for the chosen modality will automatically open	

Step 3	Complete CRF: Change to modality:
	• Fill in all mandatory fields using the measure/legend
	• Be advised that there is one CRF for each hour of observation. This depends on the chosen RRT modality (for IHD: 0.0 hours, 4.0 hours and treatment end; for CVVH, CVVHD, CVVHDF, HVHF, CPFA: 0.0 hours, at 1.0 hour, 3.0 hours, 6.0 hours, 12.0 hours, 24.0 hours, and every 24 hours thereafter and at treatment end)
	• In the case of treatment interruption or end, specify date/time (for definition of treatment interruption or treatment end, see under 'Guidelines given in the CRF', in the text)
	• In the case of change of treatment modality after treatment interruption, fill in CRF: Criteria to modality. Then go back to the start of step 3. Once in CRF: Change to modality, do not forget to select the new modality chosen

Press 'save' and terminate CRF: Change to modality	

Step 4	At discharge, please complete CRF: Outcome

ARF, acute renal failure; CPFA, coupled plasma filtration adsorption; CRF, case report form; CVVH, continuous venovenous haemofiltration; CVVHD, continuous venovenous haemodialysis; CVVHDF, continuous venovenous haemodiafiltration; HVHF, high-volume haemofiltration; RIFLE, Risk Injury Loss of fucntion End stage renal disease; RRT, renal replacement therapy
